# High level of adherence to HAART among refugees and internally displaced persons on HAART in western equatorial region of Southern Sudan

**DOI:** 10.1186/1758-2652-13-S4-P123

**Published:** 2010-11-08

**Authors:** O Salami, A Buzu, C Nzeme

**Affiliations:** 1 Tambura hospital/ International Medical corps, Yambio, Sudan

## Background

Signature of the Comprehensive Peace Agreement (CPA) in 2005 marked the end of the 20 year war in Southern Sudan. However, the decades of war and lawlessness have completely disrupted the healthcare infrastructure in the whole of southern Sudan. HIV sero-prevalence in the western equatorial state of Southern Sudan (12.1%) is the highest in the country. the massive numbers and mobility of internally displaced persons as well as insecurity due to frequent rebel attacks make providing ART for HIV infected persons in this part of Africa very challenging.

## Objectives

This study highlights the challenges and achievements of international medical corps, with support from the WHO, in providing HAART to returning refugees and internally displaced persons in western equatorial region of Southern Sudan.

## Patients and methods

We analyzed clinic data of 159 (90 F, 69 M) adults were started on ART between July 2009 and march 2010. Most (69%) had been living in refugee camps while 12% were internally displaced persons at the time of ART commencement. 78% of patients presented with WHO stage 3 or 4 symptoms. All new patients went through a 3 day period of treatment preparation prior to ART commencement. Treatment education in the local language was done at group and individual levels during clinic visits. Songs addressing adherence were developed and used during Support group sessions to reinforce key adherence messages.

## Results

68% of patients had baseline CD4 testing prior to commencing ART. Mean baseline CD4 count was 97cells/uL. All patients are presently on first line HAART. 65% of patients were started on AZT/3TC/NVP, 20% on AZT/3TC/EFV, and 15% on D4T/3TC/NVP. The commonest side effect observed were anaemia (6%), skin rash (4%), and gastro-intestinal discomfort (3.5%).

Of the 102 patients who had taken HAART for at least 6 months, 88% reported adherence levels of >95% (had missed less than 3 doses within last month). Adherence was higher in females (92%) compared to males (80%). Of those who reported missing more than 3 doses, 71% gave rebel attacks as the reason they were unable to return to the clinic for their drug pick-ups.

## Conclusions

Despite challenges related to insecurity in Southern Sudan, successful antiretroviral therapy can be provided. Good level adherence remains an important determinant of success of ART and efforts must be made to institute comprehensive treatment education as a key strategy especially in resource limited settings.

